# Standard 12 month dialectical behaviour therapy for adults with borderline personality disorder in a public community mental health setting

**DOI:** 10.1186/s40479-017-0070-8

**Published:** 2017-09-23

**Authors:** Daniel Flynn, Mary Kells, Mary Joyce, Paul Corcoran, Conall Gillespie, Catalina Suarez, Mareike Weihrauch, Padraig Cotter

**Affiliations:** 1 Cork Mental Health Services, Health Service Executive, Block 2, St Finbarr’s Hospital, Cork, Ireland; 2 National Suicide Research Foundation, Western Gateway Building, University College Cork, Cork, Ireland; 3 Department of Epidemiology and Public Health, Western Gateway Building, University College Cork, Cork, Ireland

**Keywords:** Borderline personality disorder, Dialectical behaviour therapy, Adults, Effectiveness, Public health service, Community settings

## Abstract

**Background:**

Dialectical behaviour therapy (DBT) is noted to be an intervention with a growing body of evidence that demonstrates its efficacy in treating individuals diagnosed with borderline personality disorder (BPD). Evidence for the effectiveness of DBT in publicly funded community mental health settings is lacking however. No study to our knowledge has been published on the effectiveness of a 12 month standard DBT programme without adaptations for individuals with BPD in a publicly funded community mental health setting and no study has included data across multiple time-points. The main objective of the current study was to determine if completion of a 12 month DBT programme is associated with improved outcomes in terms of borderline symptoms, anxiety, hopelessness, suicidal ideation, depression and quality of life. A secondary objective includes assessing client progress across multiple time-points throughout the treatment.

**Methods:**

Fifty-four adult participants with BPD completed the standard DBT programme across four sites in community mental health settings in the Republic of Ireland. Data was collected by the DBT therapists working with participants and took place at 8 week intervals across the 12 month programme. To explore the effects of the intervention for participants, linear mixed-effects models were used to estimate change utilising data available from all time-points.

**Results:**

At the end of the 12 month programme, significant reductions in borderline symptoms, anxiety, hopelessness, suicidal ideation and depression were observed. Increases in overall quality of life were also noted. In particular, gains were made during the first 6 months of the programme. There was a tendency for scores to slightly regress after the six-month mark which marks the start of the second delivery of the group skills cycles.

**Conclusions:**

The current study provides evidence for the effectiveness of standard DBT in publicly funded community mental health settings. As participants were assessed at the end of every module, it was possible to observe trends in symptom reduction during each stage of the intervention. Despite real-world limitations of applying DBT in community settings, the results of this study are comparable with more tightly controlled studies.

**Trial registration:**

ClinicalTrials.gov ID: NCT03166579; Registered May 24th 2017 ‘retrospectively registered’

**Electronic supplementary material:**

The online version of this article (doi:10.1186/s40479-017-0070-8) contains supplementary material, which is available to authorized users.

## Background

Borderline personality disorder (BPD) is a mental health diagnosis characterised by a pervasive pattern of instability of interpersonal relationships, self-image, affect, and marked impulsivity [1]. BPD typically features patterns of cognitive, emotional and behavioural dysregulation that often manifests in self-harm and suicidal behaviours [2]. It is estimated that the prevalence of BPD in the general population is between 0.7 and 1% [3–5]. Up to 20% of psychiatric inpatients are estimated to have this disorder and it is diagnosed predominantly (about 75%) in females [1]. In the Republic of Ireland, it is estimated that BPD is a feature of 11–20% of clinical presentations to outpatient clinics within mental health services [6]. This is similar to what has been reported in other countries including the United Kingdom [7], North America [8] and other parts of Europe (e.g. Denmark; [9]).

One of the most researched interventions for treating BPD is dialectical behaviour therapy (DBT; [10–12]). A dialectical philosophy and the biosocial theory underlie DBT and guide the functions and modes of therapy used throughout this 12 month programme [13]. Dialectics are positions which appear to be in conflict although both are true, i.e. wanting life to be better but continuing to engage in behaviours that cause one harm [11]. Biosocial theory posits that certain biological and temperamental vulnerabilities coupled with a perceived invalidating environment result in emotional and behavioural dysregulation [11].

“Standard” DBT is delivered by a team of multidisciplinary mental health professionals and comprises of individual therapy sessions for each patient, group skills training sessions, phone coaching and consultation meetings for the clinicians on the DBT team [11]. There are four DBT group skills training modules: mindfulness, distress tolerance, emotion regulation and interpersonal effectiveness. Group skills are delivered in blocks of three 8-week cycles which teach mindfulness in the first 2 weeks of each cycle followed by 6 weeks each of distress tolerance, emotion regulation and interpersonal effectiveness. The three cycles are delivered over a 24-week period and are then repeated. DBT has five functions: augmenting behavioural capabilities; generalising gains to the natural environment; improving motivation; structuring environment to reinforce functional as opposed to dysfunctional behaviour and improving therapist motivation and capabilities [13].

There is a growing body of evidence that demonstrates the efficacy of DBT in treating individuals diagnosed with BPD. To date, more than a dozen randomised controlled trials (e.g. [14–16]) have investigated the efficacy of DBT at multiple independent sites [17, 18]. Participation in DBT has been found to be associated with reductions in a range of difficulties found amongst participants including suicidal behaviour [15, 19–21], suicidal ideation [22, 23], BPD symptoms [24], hopelessness [22] and depression [23, 24]. It has also been associated with improved adjustment [20] and quality of life [21, 24], as well as reduced health service utilisation and/or inpatient psychiatric days [19, 21, 23, 24]. A recent systematic review of randomised studies has shown that DBT is significantly better than treatment as usual in terms of leading to reductions in self-harm, decreases in ineffective expression of anger and improvement in general functioning [25].

While the outlined research studies have demonstrated the efficacy of DBT in treating BPD in controlled settings, there is a dearth of published research reporting on the effectiveness of DBT in publicly funded community mental health settings. Comtois, Elwood, Holdraft, Smith and Simpson [26] conducted the first effectiveness study of DBT in a community mental health setting. Their study involved a number of adaptations to standard DBT: weekly skills groups were delivered in two 90-min sessions and the study trial also offered individual DBT case management. The few other studies that have been conducted in community settings have focused on a 6 month DBT programme (e.g. [27, 28]) or have reported on cluster B personality presentations including but not focusing exclusively on BPD [29]. A number of studies have also highlighted limitations with regard to small sample sizes (e.g. [30, 31]).

### Current study

In the Republic of Ireland, an Expert Group on Mental Health Policy published a government policy framework for publicly funded community mental health services [6] which recommended DBT as an evidence-based treatment for people with BPD [32]. This report outlined that a dedicated DBT team should be established in each catchment area (300,000 population) across the Irish public health service (Health Service Executive). In line with these recommendations, clinicians from four community mental health teams in a region in Ireland completed training in DBT between 2010 and 2012. As there was no study to our knowledge which documented the effectiveness of a 12-month standard DBT programme without adaptations in a publicly funded community mental health setting, we decided to rigorously evaluate the programme to identify if this intervention would be effective in treating adults with BPD. Therefore, the current study investigates the use of DBT (a 12 month standard programme) as a treatment for individuals with BPD in public community mental health settings. The main objective is to determine if completion of a 12 month standard DBT programme is associated with improved outcomes in terms of borderline symptoms, anxiety, hopelessness, suicidal ideation, depression and quality of life. As no study to our knowledge has included data collection at each eight-week cycle during the programme, a secondary objective includes the assessment of client progress across multiple time-points throughout the treatment.

## Methods

### Design and study setting

In Ireland, DBT is typically delivered in community based mental health settings in the public health service [32]. Within this context, core multi-disciplinary staff from multiple community mental health teams come together to train in DBT and offer this intervention as an evidence-based treatment for individuals with BPD in their local mental health service. Therefore, participants in this study were treatment seeking individuals who were attending their local adult community mental health service.

Clients were referred to one of four DBT programmes in the southern region of Ireland by their community mental health team. Clients were subsequently screened by a member of the DBT team in their area to identify suitability for treatment. All clients had a history of self-harm behaviour, and current emotion and behavioural dysregulation. If DBT was deemed suitable for the client, they were invited to engage in pre-treatment which typically consisted of up to 6 sessions prior to engaging with all treatment modalities. If clients started the DBT programme, they were invited to participate in the research evaluation. No compensation was available to participants for partaking in this study. Recruitment of participants took place from August 2010 to July 2013. All individuals who were approached consented to participate in the research evaluation resulting in 100% participation rate for this study. Ethical approval to conduct this research study was received from the Clinical Research Ethics Committee of the Cork Teaching Hospitals.

### Participants

There were 71 participants in this study, consisting of 61 females and 10 males. Participants were seeking treatment in their local publicly funded community mental health service. Participants ranged in age from 19 to 56 (mean = 40, SD = 9.76). To be included in the research study, participants were required to meet criteria for either borderline personality disorder (DSM-IV-TR) or emotionally unstable personality disorder (ICD-10). Table 1 summarises the sample characteristics of participants.

**Table 1:** Sample characteristics of participants (*N* = 71)

Characteristics	Number	Percent
*Gender*		
Female	61	86
Male	10	14
*Age*		
18–24	16	23
25–34	23	32
35–44	20	28
45–54	11	16
55–64	1	1
65+	0	0
*Employment Status*		
Employed	15	21
Unemployed	27	38
Student	6	9
Other	4	6
Did not specify	19	27
*Relationship Status*		
Single	35	49
In a relationship	1	1
Married	14	20
Separated/ Divorced	10	14
Did not specify	11	16

### Therapists and treatment

All four DBT teams in this study were newly established teams who had undertaken Intensive Training™ with a licensed training provider (British Isles DBT Training). The teams undertook training between April 2010 and May 2012. Each of the DBT teams across four sites comprised of four to ten multi-disciplinary staff members representing psychiatry, clinical psychology, nursing, art therapy and social work.

Each of the DBT teams delivered the standard DBT programme as described in Cognitive-Behavioural Treatment of Borderline Personality Disorder [11] and Training Manual for Treating Borderline Personality Disorder [12]. The DBT programme was delivered over a 12 month period and included weekly individual therapy sessions for each participant, weekly group skills training sessions delivered by two DBT therapists (leader and co-leader), phone coaching (as per individual therapist limits) and weekly consultation meetings for the therapists on the DBT team.

Expert supervision was provided to all four teams by experienced DBT supervisors in the U.K. and U.S.A. One of the four teams had access to supervision from January 2012 and the other three teams had supervision available from May 2012. Supervision was dependent on supervisor availability and varied in frequency from weekly to quarterly.

### Measures

Effectiveness of the programmes was measured using the following outcome measures:

*Borderline Symptom List* (BSL-23; [33]). The BSL-23 comprises 23 items measuring borderline-typical symptomatology. Bohus et al.’s [33] results on assessing the scales properties demonstrated that the BSL-23 has high internal and test-retest reliability. In the current study, the internal reliability for the BSL was .92.

*Beck Anxiety Inventory* (BAI; [34]). The BAI is a 21 item self-report multiple choice survey which evaluates both physiological and cognitive symptoms of anxiety. A meta-analysis reviewing the BAI from 192 studies found it to demonstrate good internal consistency, test-retest reliability and structural validity [35]. In the current study, the internal reliability of the BAI at baseline was .92.

*Beck Hopelessness Scale* (BHS; [36]). The BHS is a 20 item self-report measure which assesses key aspects of hopelessness. The BHS has demonstrated good reliability and validity in psychiatric samples [37]. In the current study, the internal reliability for the BHS at baseline was .89.

*Beck Scale for Suicide Ideation* (BSS; [38]). The BSS is a 21 item assessment developed to identify individuals at risk of suicide. Items are rated on a scale of 0 to 3 with only the initial 19 items used to compute total scores. The BSS has both high internal consistency and validity [39]. The internal reliability of the BSS was .94 in the current study.

*Beck Depression Inventory – Second Edition* (BDI-II; [40]). The BDI-II is a 21 item self-report measure of symptoms and attitudes related to depression. The BDI-II has demonstrated strong psychometric properties [41]. In the current study, the internal reliability for the BDI-II at baseline was .88.

*World Health Organisation Quality of Life Questionnaire* (WHOQOL-BREF; [42]). The WHOQOL-BREF was developed collaboratively in a number of centres to act as an international cross-culturally comparable quality of life assessment. It comprises 26 items across four domains: physical health (domain 1); psychological health (domain 2); social relationships (domain 3); and environment (domain 4). A cross-sectional study conducted across 23 countries demonstrated that the measure has good to excellent psychometric properties of reliability and is a valid assessment of quality of life [43]. The internal reliability for the domains listed above were .78, .70, .62 and .80 respectively.

### Procedure

Data collection was completed by the DBT therapists working with participants in the research study was obtained by the DBT therapist who carried out the initial screening appointment and pre-treatment with each individual participant. Data collection took place at 8 week intervals across the 12 month programme. There were seven time-points for data collection: baseline (T1- start of programme); 2 months (T2); 4 months (T3); 6 months (T4); 8 months (T5); 10 months (T6); 12 months (T7). Baseline measures were typically completed in an individual session with the participant’s DBT therapist prior to starting the full programme i.e. individual therapy and group skills. Measures at subsequent time-points were typically completed in the group skills sessions. If a participant was not present at the group skills session, their DBT therapist was asked to administer the measures in the next individual therapy session.

### Statistical analysis

All outcome measures were quantitative and were summarised by their mean and standard deviation. T-tests and analyses of variance were used to assess baseline differences in the outcome measures by gender, age group and site of study. To explore the effects of the intervention on participants, linear mixed-effects models were used to estimate change utilising data available from all time-points. These models adjusted for clustering in the data due to repeated measures on the same individuals and the intervention being delivered at four sites. Data were analysed using Stata version 13.1 for Windows.

## Results

The means and standard deviation for each of the nine outcome measures at each time-point are detailed in Table 2. At baseline, there were no notable differences in these measures when examined by gender, age group and study site, with the exception of scores on the BHS across sites (see Additional file 1).

**Table 2:** Outcome measure means (M) and standard deviations (SD) at each study time-point

Variable	T1 M (SD) *n* = 66	T2 M (SD) *n* = 56	T3 M (SD) *n* = 45	T4 M (SD) *n* = 51	T5 M (SD) *n* = 42	T6 M (SD) *n* = 37	T7 M (SD) *n* = 44
BSL	59.78 (19.13)	51.43 (25.29)	41.95 (25.27)	41.18 (23.93)	42.93 (24.30)	36.71 (25.62)	36.24 (26.16)
BAI	29.65 (13.74)	29.79 (15.06)	24.57 (13.98)	22.99 (12.82)	22.39 (14.21)	22.50 (13.40)	19.53 (13.11)
BHS	13.76 (4.98)	12.24 (5.68)	11.28 (6.33)	10.14 (6.47)	9.63 (6.25)	8.71 (6.45)	8.17 (6.44)
BSS	14.17 (9.65)	11.94 (9.32)	8.63 (10.30)	10.85 (10.08)	9.44 (10.98)	7.59 (9.13)	7.29 (9.57)
BDI	38.33 (10.96)	31.27 (14.50)	25.58 (14.82)	27.14 (14.71)	26.39 (16.83)	23.57 (16.63)	22.82 (16.43)
QoL Physical Health	11.33 (3.09)	11.77 (3.08)	12.60 (3.30)	11.75 (3.30)	12.68 (3.30)	13.33 (3.51)	13.16 (3.07)
QoL Psych. Health	7.58 (2.40)	8.11 (2.64)	9.36 (3.39)	9.13 (3.35)	10.27 (3.79)	10.60 (4.20)	10.36 (3.60)
QoL Soc. Relationship	10.04 (3.64)	9.89 (3.56)	11.23 (3.66)	10.70 (3.96)	10.58 (4.05)	12.74 (4.12)	12.23 (4.25)
QoL Environment	11.89 (3.04)	11.86 (3.05)	13.06 (3.49)	12.64 (3.22)	13.38 (3.13)	13.79 (3.41)	13.61 (3.20)

Based on the data available at each time-point, there was evidence of decreases in BSL, BAI, BHS, BSS and BDI scores, and increases in quality of life domain scores (see Table 2). This was confirmed by the linear mixed-effects models. As detailed in Table 3, there were highly statistically significant changes in all nine outcome measures. The most marked changes both in terms of immediacy and magnitude were evident in relation to the BSL, BHS, BSS and BDI. On average, scores on these measures decreased by 41 to 49% over the duration of the programme.

**Table 3:** Outcome measure estimated baseline means (M) and changes at subsequent time-points

	Estimated baseline M (95% CI)	Change at T2 M (95% CI)	Change at T3 M (95% CI)	Change at T4 M (95% CI)	Change at T5 M (95% CI)	Change at T6 M (95% CI)	Change at T7 M (95% CI)	% change
BSL	58.11 (52.26, 63.96)	−8.57** (−14.64, −2.49)	−12.65*** (−18.99, −6.3)	−16.28*** (−22.74, −9.82)	−13.2*** (−19.84, −6.56)	−20.69*** (−27.53, −13.84)	−24.46*** (−30.98, −17.95)	−42
BAI	29.75 (26.49, 33.01)	−0.51 (−3.64, 2.63)	−4* (−7.32, −0.68)	−6.64*** (−9.85, −3.43)	−4.75** (−8.19, −1.32)	−5.98*** (−9.48, −2.48)	−9.43*** (−12.7, −6.17)	−32
BHS	13.74 (11.7, 15.79)	−2.2** (−3.58, −0.81)	−2.54*** (−4.08, −0.99)	−4.22*** (−5.65, −2.79)	−3.97*** (−5.5, −2.44)	−4.71*** (−6.23, −3.19)	−5.61*** (−7.07, −4.15)	−41
BSS	14.29 (11.87, 16.71)	−3.25** (−5.69, −0.8)	−4.71*** (−7.31, −2.11)	−3.7** (−6.24, −1.17)	−5.07*** (−7.73, −2.41)	−6.34*** (−9.01, −3.68)	−7.03*** (−9.61, −4.45)	−49
BDI	38.37 (34.89, 41.84)	−7.27*** (−10.35, −4.19)	−11.11*** (−14.32, −7.89)	−11.57*** (−14.65, −8.48)	−12.27*** (−15.54, −9)	−15.15*** (−18.55, −11.74)	−17.03*** (−20.42, −13.64)	−44
QoL Phys.	11.67 (10.85, 12.49)	0.03 (−0.66, 0.72)	0.59 (−0.13, 1.32)	0.12 (−0.58, 0.82)	0.52 (−0.26, 1.29)	1.18** (0.44, 1.93)	1.11** (0.39, 1.82)	+10
QoL Psych.	7.9 (7.1, 8.69)	0.21 (−0.52, 0.94)	1.01** (0.25, 1.78)	1.32*** (0.58, 2.06)	1.87*** (1.05, 2.68)	2.45*** (1.67, 3.23)	2.5*** (1.75, 3.25)	+32
QoL Soc.	10.11 (9.13, 11.09)	−0.32 (−1.39, 0.74)	0.86 (−0.26, 1.98)	0.39 (−0.69, 1.47)	0.62 (−0.57, 1.81)	2.4*** (1.26, 3.54)	2.14*** (1.04, 3.23)	+21
QoL Envir.	12.02 (11.22, 12.82)	−0.13 (−0.78, 0.51)	0.64 (−0.04, 1.32)	0.42 (−0.24, 1.08)	1.02** (0.29, 1.75)	1.3*** (0.61, 2)	1.3*** (0.63, 1.97)	+11

* = *p* < .05; ** = *p* < .01; *** = *p* < .001

Changes at each follow-up are relative to the baseline; 95% CI = 95% confidence interval

**Fig.1:**
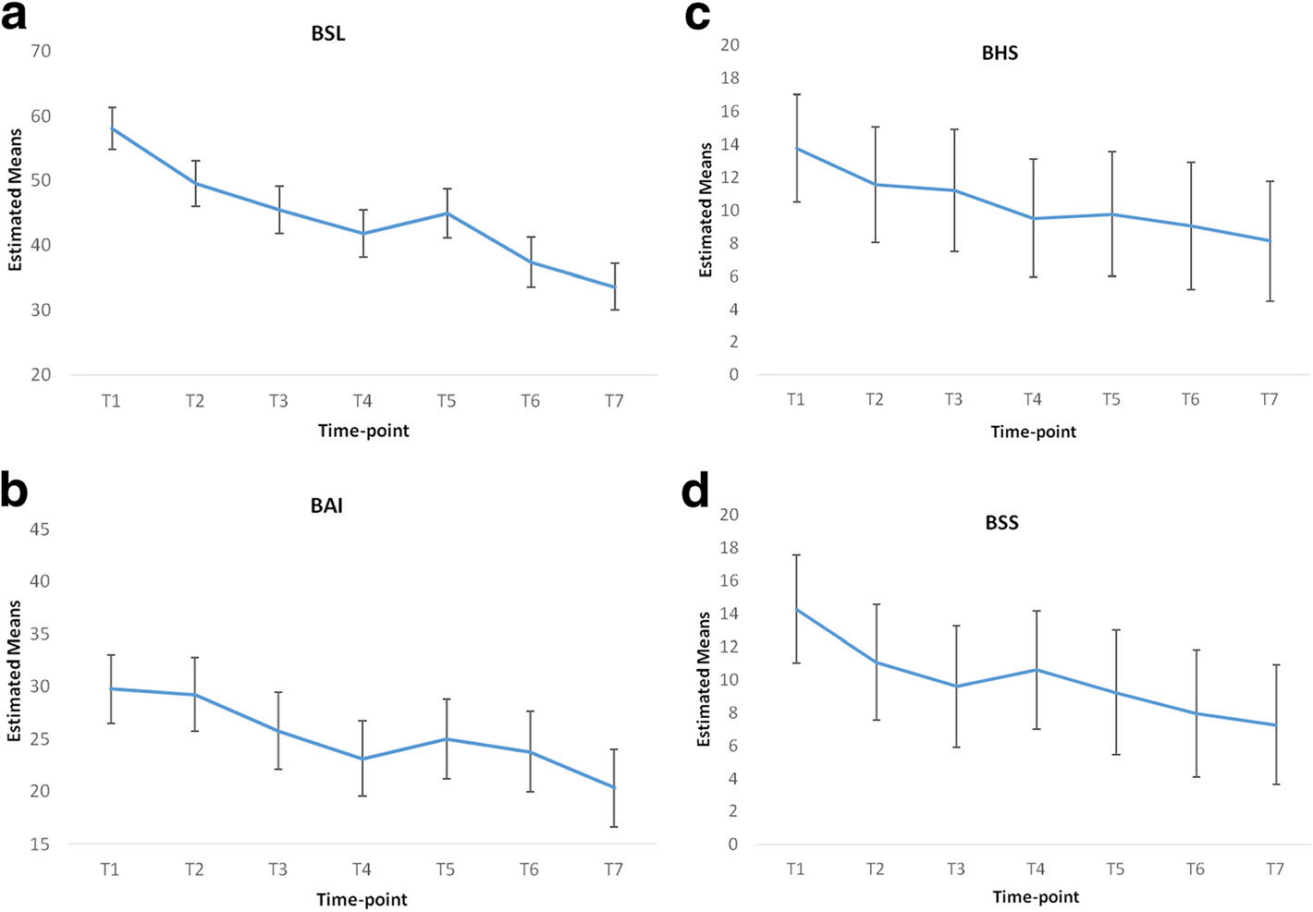
Adjusted means and confidence intervals at each time-point for: **a** BSL **b** BAI **c** BHS **d** BSS

Scores on the BSL, BAI and BHS gradually decreased from T1 to T4 which represents the half-way point of the programme. At T5, a slight increase in scores on these measures was observed, but all scores continued to decrease once again from T5 to T7. This information is highlighted in Fig. 1. Scores on the BSS followed a similar trend to that of the BSL, BAI and BHS, although the regression towards baseline for the BSS occurred at T3 rather than T4 as with the other measures.

**Fig. 2:**
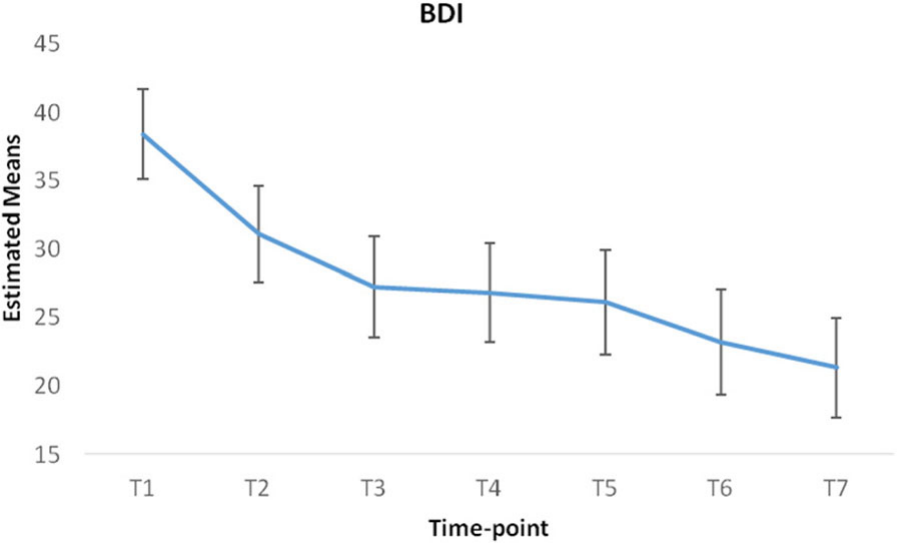
Adjusted means and confidence intervals at each time-point for BDI

Scores on the BDI gradually decreased at each time-point from T1 to T7 as illustrated in Fig. 2 although the rate of improvement reduced at the mid-point of the programme (T4).

**Fig. 3:**
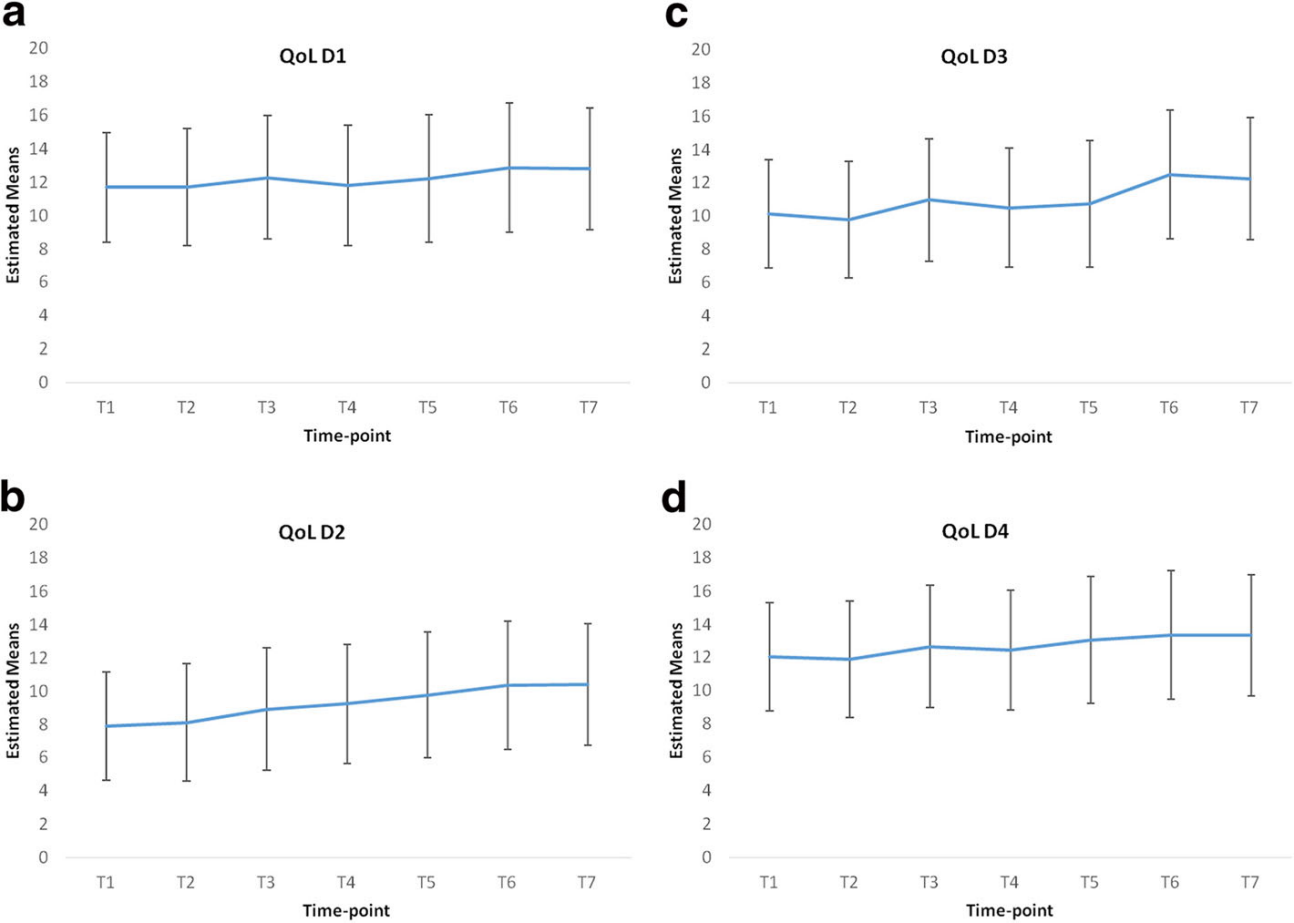
Adjusted means and confidence intervals at each time-point for: **a** QoL D1 **b** QoL D2 **c** QoL D3 **d** QoL D4

All four domains of the quality of life measure increased significantly from T1 to T7; the most relevant domain for participants in this study, and the domain that had the greatest increase in scores with an increase on average by 32% over the course of the programme was domain 2 which is the psychological health domain (Fig. 3b).

**Fig. 4:**
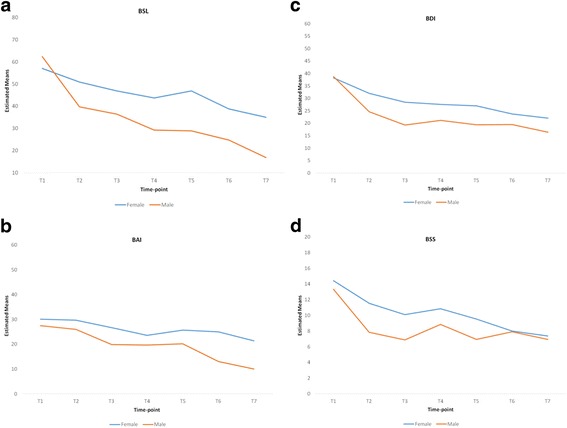
Adjusted means for males and females at each time-point for: **a** BSL **b** BAI **c** BDI **d** BSS

Male participants experienced earlier gains and showed greater overall decreases in BSL, BAI, BDI and BSS scores (Fig. 4). However, with only 10 male participants, this study did not have the power to demonstrate that these gender differences were statistically significant.

## Discussion

This was the first study to investigate the effectiveness of a 12 month standard DBT programme without adaptations across multiple time-points in a publicly funded community mental health setting. The current study found that DBT was associated with significant reductions in levels of borderline symptoms, anxiety, hopelessness, suicidal ideation and depression. Increases in overall quality of life were also observed. These improvements were similar to the results of other DBT effectiveness studies. Similar to Prendergast and McCausland [31], depression scores as measured by the BDI were in the ‘severe’ range at the start of treatment. These scores had significantly reduced to the lower end of the ‘moderate’ range by the end of the intervention. Similar findings can be found on the measure of suicidality (BSS) which observed similar scores at baseline to Pasieczny and Connor [28] with scores decreasing by almost half by the end of the intervention.

As participants were assessed at the end of every module, it was possible to observe trends in symptom reduction during each stage of the intervention. In particular, gains were made during the first 6 months of the programme. This ‘early treatment response’ or ‘sudden gain’ has been shown to be a potential mediator of change in other therapies for other difficulties such as depression treated by cognitive behaviour therapies (e.g. [44, 45]). To our knowledge, this is the first time such a trend has been observed amongst this population in response to a DBT intervention.

There was also a tendency for scores to slightly regress after the 24 week point which marks the start of the second delivery of the skills cycles. It is difficult to provide a concrete explanation for this mild increase in suicidal ideation, borderline symptoms, anxiety and hopelessness at this point. One possible explanation is that client functioning did not match client’s expectation of progress after completion of the first delivery of the skills modules. It is possible that clients may hold the perception that their gains should be greater at this point which may manifest as client anxiety at the mid-point of the programme. Therapist feedback would indicate that the mid-point of the programme can sometimes present as a challenge with regard to motivation. These results warrant further investigation however as the proposed explanations are merely speculative. It is also important to note that psychological health and mood were not affected during this period and this slight regression was temporary. A significant decrease in scores was observed again at T6 and T7 (the end of the intervention).

This study highlighted a potential difference between males and females in terms of gains made during the intervention although observed differences were not statistically significant. It is possible that if the number of males in this study were greater, these differences might have yielded statistical significance. Given that much of the published research has focused primarily on female populations (e.g. [26, 46]), this warrants further consideration and exploration in studies with larger numbers of male participants.

It should be noted that supervision for teams was not available from the first intake of programme participants at each of the four sites. Despite limited and late availability of supervision, and variations in the quantity of supervision utilised, DBT participants across the four sites made significant gains during the course of the intervention. It is recognised that supervision is fundamental in enhancing therapists’ motivation, competence consolidation, adherence to the model, and ultimately sustainability of DBT programmes [47]. However, it is acknowledged that financial constraints, availability of expert DBT supervisors, and scheduling and logistical constraints of linking with supervisors in other jurisdictions and time zones can pose significant barriers to effectively engaging in and benefitting from expert supervision. In view of these constraints, it would be useful for future research to consider the relationship between quantity of supervision and client outcomes.

### Limitations

One of the challenges of working in a publicly funded mental health system is that clinicians have a responsibility to treat every individual who presents to their service. It is not possible to stream people into an intervention group versus a control group where no intervention is given when best practice guidelines (e.g. NICE [48]) and a large body of published international research indicates the effectiveness of an intervention such as DBT for treating this client group. At the time of this study, there was no alternative evidence-based intervention available that could have been used as a comparison group at any of the four sites. Once the evidence-based treatment (in this case, DBT) was available in an area, it was necessary to offer individuals the intervention where it was indicated. Therefore, no control group could be accessed for inclusion in this study. Thus, a limitation of this study is that it uses an uncontrolled, non-randomised design making it difficult to determine whether changes that were found were wholly due as a result of treatment, or in part, related to other factors such as the passage of time. Future research would benefit from exploring the impact of this intervention in comparison to treatment-as-usual, or other evidence-based interventions for BPD in publicly funded community based settings.

While the outcome measures used in this study were based on international research on DBT, service utilisation data regarding emergency department visits and psychiatric inpatient admissions was not collected across the four sites. Although it was possible to establish the effectiveness of the 12 month standard DBT programme with regard to clinical outcomes for DBT participants, it was not possible to evaluate the effectiveness of the programme in terms of history of self-harm behaviour and cost effectiveness. Previous research (e.g. [24]) highlight significant costs associated with BPD, therefore future research might explore the costs incurred for individuals with BPD attending publicly funded community mental health services.

As this study was carried out in a publicly funded community mental health setting with limited resources, there was no dedicated research team to co-ordinate the research evaluation. The data collected for this study was thus gathered by the DBT therapists at each of the four study sites. Therapists’ administration of measures to individual clients may introduce an experimenter bias as it is possible that DBT participants will respond to measures in a way that they hope will please their therapist. The lack of a supporting research team also resulted in measures not being administered within the recommended timeframe or data collection being missed on occasion. This resulted in incomplete datasets at some time-points in this study. These limitations, alongside other challenges such as the lack of electronic records and computerised data collection tools, highlight the challenges of conducting research in a real-world setting.

It is also acknowledged that a further limitation of the study is the absence of adherence coding for therapists providing the intervention. Financial and practical constraints of conducting adherence rating in a publicly funded mental health system resulted in it being beyond the scope of this study.

### Future directions

The results of this study provide evidence for the effectiveness of standard DBT in community settings. The intervention was provided directly to the client by the therapist without reference to treating the wider system in which the individual resides. There is promising research evidence to support benefits of family interventions such as Family Connections [49] and how systemic work could further moderate change for individuals with BPD. This has the potential to further enhance the effectiveness of the DBT treatment. Given that this study is limited to one geographical area in Ireland, future research will offer further insight into the utility of DBT across the wider national public health system. Given that the sites in this study were independent of each other, further investigations could consider the benefits of a more co-ordinated approach to national implementation addressing some of the potential challenges noted in the growing field of implementation science research [32].

## Conclusion

The current study provides evidence for the effectiveness of standard DBT in publicly funded community mental health settings. Despite real-world limitations of applying DBT in public community settings, the results of this study are comparable with more tightly controlled studies. Although this study took place across four sites with four different teams delivering the treatment, significant gains were made for the entire study sample. We hope that the results of this study will encourage clinicians working in publicly funded community mental health settings to implement standard DBT programmes to meet the needs of this client group.

## Additional file

Additional file 1: Table A1. Mean, standard deviation and significance level for males and females at baseline on nine outcome measures. **Table A2.** Mean, standard deviation and significance level for four age groups at baseline on nine outcome measures. **Table A3.** Mean, standard deviation and significance level for four study sites at baseline on nine outcome measures. (DOCX 15 kb)

## Abbreviations

BAI: Beck anxiety inventory
BDI: Beck depression inventory
BHS: Beck hopelessness scale
BPD: Borderline personality disorder
BSL: Borderline symptom list
BSS: Beck scale for suicide ideation
CBT: Cognitive behaviour therapy
DBT: Dialectical behaviour therapy
NICE: National institute for health and care excellence
QOL: Quality of life
