# Supplementary material for: Standard 12 month dialectical behaviour therapy for adults with borderline personality disorder in a public community mental health setting
Source: Borderline Personal Disord Emot Dysregul. 2017 Sep 23;4:19. doi: 10.1186/s40479-017-0070-8 (PMC5610433; doi:10.1186/s40479-017-0070-8)
Supplement: Additional file 1: Table A1. — Mean, standard deviation and significance level for males and females at baseline on nine outcome measures. Table A2. Mean, standard deviation and significance level for four age groups at baseline on nine outcome measures. Table A3. Mean, standard deviation and significance level for four study sites at baseline on nine outcome measures. (DOCX 15 kb) [file 40479_2017_70_MOESM1_ESM.docx]

Additional file

**Table A.1**

Mean, standard deviation and significance level for males and females at baseline on nine outcome measures

| Variable | Group | |  |
| --- | --- | --- | --- |
|  | Male  *M* (SD) | Female  *M* (SD) | *p* |
| BSL | 62.40 (20.23) | 59.27 (19.07) | .64 |
| BAI | 27.45 (14.47) | 30.05 (13.71) | .59 |
| BHS | 13.40 (5.89) | 13.83 (4.86) | .80 |
| BSS | 14.00 (12.20) | 14.20 (9.27) | .96 |
| BDI | 38.70 (9.72) | 38.26 (11.28) | .91 |
| QoL D1 | 12.00 (3.13) | 11.25 (3.10) | .58 |
| QoL D2 | 9.44 (3.22) | 7.34 (2.21) | .04 |
| QoL D3 | 10.44 (3.62) | 9.99 (3.68) | .77 |
| QoL D4 | 13.42 (4.13) | 11.70 (2.88) | .20 |

**Table A.2**

Mean, standard deviation and significance level for four age groups at baseline on nine outcome measures

| Variable | Group | | | | |
| --- | --- | --- | --- | --- | --- |
|  | 18-24  *M* (SD) | 25-34  *M* (SD) | 35-44  *M* (SD) | 45-54  *M* (SD) | *p* |
| BSL | 56.07 (15.89) | 65.28 (17.25) | 58.17 (20.99) | 61.28 (22.47) | .42 |
| BAI | 28.74 (11.18) | 28.15 (15.84) | 31.88 (14.46) | 31.35 (11.95) | .63 |
| BHS | 14.53 (3.72) | 13.29 (6.03) | 13.35 (5.07) | 14.35 (5.06) | .94 |
| BSS | 19.98 (5.90) | 11.53 (9.62) | 12.24 (9.96) | 16.33 (11.14) | .12 |
| BDI | 38.31 (8.34) | 39.09 (13.70) | 36.89 (11.25) | 40.36 (10.02) | .92 |
| QoL D1 | 12.53 (2.57) | 11.18 (2.95) | 10.84 (3.82) | 10.54 (2.74) | .55 |
| QoL D2 | 7.90 (2.83) | 7.57 (3.16) | 7.48 (1.64)) | 7.23 (1.70) | .98 |
| QoL D3 | 10.46 (3.80) | 9.96 (3.70) | 9.87 (3.70) | 9.93 (4.01) | .99 |
| QoL D4 | 12.42 (2.91) | 11.45 (3.19) | 12.15 (3.07) | 11.30 (3.42) | .87 |

**Table A.3**

Mean, standard deviation and significance level for four study sites at baseline on nine outcome measures

| Variable | Group | | | | |
| --- | --- | --- | --- | --- | --- |
|  | Site 1  *M* (SD) | Site 2  *M* (SD) | Site 3  *M* (SD) | Site 4  *M* (SD) | *p* |
| BSL | 57.40 (17.06) | 68.50 (15.92) | 62.34 (32.30) | 58.71 (19.47) | .48 |
| BAI | 29.49 (13.63) | 35.66 (13.58) | 33.17 (17.45) | 25.05 (12.13) | .29 |
| BHS | 14.78 (4.78) | 15.62 (3.03) | 14.12 (3.83) | 9.79 (5.29) | .01 |
| BSS | 14.81 (9.41) | 15.80 (7.42) | 14.17 (3.06) | 11.36 (13.51) | .72 |
| BDI | 38.63 (11.17) | 40.45 (8.86) | 40.14 (8.67) | 34.70 (13.26) | .64 |
| QoL D1 | 11.85 (2.93) | 10.60 (3.33) | 9.25 (1.82) | 11.77 (3.62) | .24 |
| QoL D2 | 7.84 (2.56) | 6.74 (2.37) | 6.82 (1.16) | 8.04 (2.52) | .50 |
| QoL D3 | 10.10 (3.95) | 9.33 (3.53) | 9.33 (2.67) | 10.93 (3.60) | .77 |
| QoL D4 | 12.72 (3.23) | 10.33 (3.53) | 10.79 (1.94) | 11.62 (1.93) | .15 |
